# Public Health Response to Toxigenic Respiratory Diphtheria Outbreaks at Correctional Facility, South Africa, 2023–2025

**DOI:** 10.3201/eid3206.251957

**Published:** 2026-06

**Authors:** Maria Jose, Nectarios Papavarnavas, Arifa Parker, Charlene Lawrence, Janine Bezuidenhout, Levani Naidoo, Yolanda Cottee, Kathryn Grammer, Wentzel Dowling, Jocelyn Moyes, Anne von Gottberg, Mignon du Plessis, Hassan Mahomed

**Affiliations:** Western Cape Department of Health and Wellness, Cape Town, South Africa (M. Jose, N. Papavarnavas, A. Parker, C. Lawrence, J. Bezuidenhout, L. Naidoo, Y. Cottee, H. Mahomed); University of Cape Town, Cape Town (M. Jose, N. Papavarnavas, W. Dowling); Stellenbosch University, Cape Town (A. Parker, K. Grammer, H. Mahomed); National Health Laboratory Service at Groote Schuur Hospital, Cape Town (W. Dowling); National Institute for Communicable Diseases, Johannesburg, South Africa (J. Moyes, A. von Gottberg, M. du Plessis); University of the Witwatersrand, Johannesburg (A. von Gottberg, M. du Plessis)

**Keywords:** diphtheria, respiratory infections, bacteria, carceral health, congregate setting, correctional facility, toxigenic, Corynebacterium diphtheriae, outbreak, adult vaccines, vaccine hesitancy, public health, South Africa

## Abstract

Since 2023, there have been 3 outbreaks of toxigenic respiratory diphtheria at a correctional facility in Cape Town, South Africa. The first outbreak in October 2023 included 1 fatal case and 8 asymptomatic carriers testing positive for *Corynebacterium diphtheriae*. In December 2024, the second outbreak resulted in 1 case and 12 asymptomatic carriers. A third outbreak in February 2025, occurring 6 weeks after the second, resulted in 1 case and 14 asymptomatic carriers among inmates and staff. Contact tracing, antimicrobial prophylaxis, and vaccination campaigns were conducted with each outbreak. We outline public health responses to the 3 outbreaks and highlight key barriers and enablers for effective control in a highly mobile, densely populated correctional setting. Effective outbreak response in correctional facilities requires intersectoral collaboration and the development of adult vaccination guidelines for staff and inmates to prevent future vaccine-preventable disease outbreaks.

Correctional service facilities are high-risk environments for the spread of infectious diseases because of overcrowded conditions, poor hygiene, reduced access to sanitation and healthcare, and lack of personal protective equipment for both inmates and staff (*1*). Although progress has been made in addressing tuberculosis and HIV among inmates, recent infectious disease outbreaks in correctional facilities, such as COVID-19 and leptospirosis, have been reported in South Africa (*2*,*3*).

Diphtheria, a category 1 notifiable medical condition in South Africa, is caused by toxin-producing strains of *Corynebacterium diphtheriae* and is known to be highly contagious and potentially fatal if not treated appropriately (*4*). The case-fatality rate for untreated, unvaccinated cases of respiratory toxigenic diphtheria has been estimated at 29%; asphyxia and endocarditis are the most frequent causes of death (*5*). Humans are the main reservoir; bacteria directly colonize the nasopharynx and spread via droplets from sneezing and coughing (*4*). Diphtheria can manifest as respiratory or cutaneous symptoms. Respiratory symptoms include low-grade fever, sore throat, and pseudomembranous pharyngitis with enlarged anterior cervical lymph nodes (bull neck). Systemic complications arising from toxin-induced tissue necrosis include myocarditis, neuropathy, nephropathy, and death (*6*). Fatalities among probable and confirmed symptomatic respiratory diphtheria cases in South Africa during January 2024–June 2025 were 12 (20%) of 60 cases (*7*). Although other countries with low vaccine coverage (Nigeria, Niger, Guinea, and Algeria) are currently experiencing diphtheria outbreaks, it is unlikely that the cases in South Africa are imported; the respiratory strains identified in South Africa to date appear to be unique to the country (*8*).

The Expanded Program for Immunisation schedule in South Africa recommends diphtheria-containing vaccine administration at 6, 10, and 14 weeks of age as the primary series, with boosters at 18 months, 6 years, and 12 years (*9*). Children <2 years of age receive diphtheria vaccination as part of the hexavalent vaccine (diphtheria, tetanus, pertussis, poliomyelitis, *Hemophilus influenzae* b, and hepatitis B). The vaccine antigen for *C. diphtheriae* is toxoid, generating antitoxin immunity. Vaccination coverage >90% is required to achieve adequate herd immunity (*5*). Booster doses at 6 and 12 years of age were given as a tetanus/diphtheria vaccine (Td); as of April 2024, the acellular pertussis vaccine (Tdap) vaccination for pregnant women became policy (*9*).

Literature on outbreak response in correctional facilities in low- and middle-income settings is limited. We describe the public health response to 3 consecutive outbreaks of toxigenic *C. diphtheriae* in a Cape Town, South Africa, correctional facility, prepared in accordance with the Canada Communicable Disease Report Outbreak reporting guide (*10*). The University of the Witwatersrand, Human Research Ethics Committee to the South Africa National Institute for Communicable Diseases, granted ethics approval (certificate no. M210752, formerly M160667) for essential communicable disease surveillance and outbreak investigation activities.

## Methods

We used the NICD and Notifiable Medical Condition definitions as a reference guide for identifying cases and contacts (*4*). We defined a confirmed case as any person meeting the clinical criteria for >1 of the clinical forms of diphtheria and laboratory confirmation of the organism and toxin production. We defined a probable case as illness in any person meeting the clinical criteria for classic respiratory diphtheria and with an epidemiologic link to a confirmed case but for whom no diphtheria testing was performed, or illness in any person meeting the clinical criteria for classic respiratory diphtheria and laboratory confirmation of the organism but for which toxin production has not been confirmed. We defined an asymptomatic carrier as a person with laboratory confirmation of the organism and toxin production but no symptoms. We defined a close contact as a person with exposure to the case or known carrier in the preceding 10 days in a household-type setting or directly exposed to respiratory secretions or large-particle droplets without appropriate personal protective equipment (PPE) or exposure to an uncovered wound of a cutaneous case without appropriate PPE. We defined close contact in a healthcare setting as a person who came into contact with the patient without wearing a fluid-repellent mask who was within 2 meters of the patient and exposed for >15 minutes. In a prison setting, sharing the same cell would be considered close contact.

### Outbreak Context

The correctional facility where the outbreaks occurred is managed by the Department of Correctional Services (DCS) and had an average number of 6,500 inmates and 1,287 staff during the period of documented outbreaks. The outbreaks occurred in the awaiting-trial section, which usually houses 1,200 inmates and 240 staff at any given time. Those awaiting trial usually have numerous contacts from court procedures and meetings with lawyers and family. Approximately 100 new and returning detainees are brought to the facility daily. The awaiting-trial section has 3 floors with 5–6 cells in use on each floor. One cell may house 60–70 detainees at a time with shared bunk beds and a sink and toilet in each.

With each confirmed outbreak (Figure), Western Cape Department of Health and Wellness (WCDHW) conducted contact tracing with symptom screening and nasopharyngeal and oropharyngeal swab testing. As a precaution, we placed detainee close contacts in group isolation and sorted them into cohorts on the basis of their symptom status initially, then reorganized cohorts after results identified confirmed cases. We offered all contacts a course of azithromycin, vaccinated them, and observed them for symptoms while awaiting their laboratory results.

**Figure:**
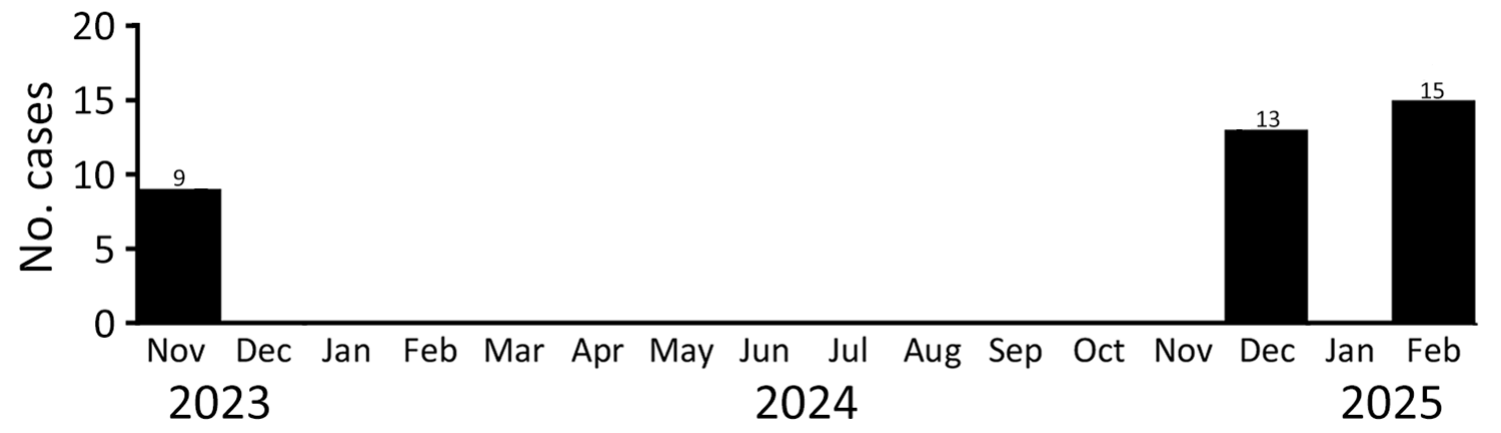
Epidemiologic curve for 3 outbreaks of toxigenic diphtheria at a correctional facility, Cape Town, South Africa, October 2023–February 2025. The outbreaks comprised 37 cases. Outbreak 1 control measures: extended vaccination campaign to staff and inmates, deep cleaning of affected section, no visitors to affected section of facility, and postponed court dates. Outbreak 2 control measure: an extended vaccine campaign to detainees in affected section of facility. Outbreak 3 control measures: extended vaccination campaign amongst detainees and staff, deep cleaning of facility, and personal protective equipment issued to visitors.

### Laboratory Methods

We cultured clinical specimens at the National Health Laboratory Service Medical Microbiology Laboratory at the local academic hospital before sending them to the national reference laboratory at NICD for confirmation and additional characterization. We immediately conducted PCR on swab specimens and positive cultures to detect the *C. diphtheriae rpoB* gene and the *tox* gene (*11*). We conducted additional species identification for cultures by matrix-assisted laser desorption/ionization time-of-flight mass spectrometry and confirmed toxin production by modified Elek test (*12*). We performed whole-genome sequencing on *C. diphtheriae* isolates to determine sequence type using the 7-locus multilocus sequence type (ST) scheme (*8*). We submitted assembled genomes to the *Corynebacterium diphtheriae* complex BIGSdb public database (https://bigsdb.pasteur.fr/diphtheria) for ST assignment and core genome multilocus ST phylogeny.

## Results

### Outbreak 1: October–November 2023

The initial diphtheria outbreak began on October 22, 2023. The index patient was a young male detainee awaiting trial who had been in the correctional facility for several months. He was admitted to the hospital on October 28 for fever, swollen neck, shortness of breath, difficulty swallowing, sore throat, and a pseudomembrane. Assessment revealed that he had renal and cardiac complications as well. He received diphtheria antitoxin (DAT) at the central hospital. He died from complications on November 5, 2023.

The case was notified to the Western Cape Department of Health and Wellness (WCDHW) provincial Communicable Diseases Control office on October 28, 2023, activating the public health outbreak response. WCDHW collaborated with DCS, NICD, National Health Laboratory Service, emergency medical services (EMS), the City of Cape Town Municipality City Health Directorate, infectious disease experts, and infection prevention and control staff. Weekly meetings ensured timely communication and kept all stakeholders up to date. Despite initial uncertainty regarding which entity would support each aspect of outbreak response, such as contact tracing and provision of vaccinations, continued engagement clarified areas of overlap.

We identified 76 close contacts of the index patient; they were DCS staff and healthcare staff who had been in close contact with him and inmates that had shared a holding area with him. We screened those contacts, provided them with postexposure vaccination and the necessary antimicrobial prophylaxis, and isolated those whose test results were pending. We had not documented how long contacts were held in a common area with the index patient at the time of the outbreak response, although that information would have been helpful to understand the extent of that exposure.

Of the 55 inmates classified as close contacts, the 8 that tested positive were assigned a separate cohort from the 47 who tested negative. Eleven inmates reported nonspecific respiratory symptoms (Table). All court dates for the inmates in the affected section were postponed. DCS staff members who were close contacts were swabbed and provided with prophylaxis and vaccine; they were placed on special paid leave and advised to isolate themselves at home until laboratory results were available within 7 days and to wear a surgical mask when in contact with household members. A doctor from the outbreak response team was in telephone contact with DCS staff daily during their home isolation to assess symptoms and provide support.

**Table:** Characteristics of 3 diphtheria outbreaks at a correctional facility, Cape Town, South Africa, October 2023–February 2025*

Characteristic	Outbreak 1, Nov 2023	Outbreak 2, Dec 2024	Outbreak 3, Feb 2025
Diphtheria contacts			
Inmate	55 (72.3)	53 (82.8)	60 (100)
Household	0	10 (15.6)	0
DCS staff	15 (19.7)	0	0
HCW	6 (7.9)	1 (1.6)	0
Total	76 (100)	64 (100)	60 (100)
Screening positivity of contacts, %	11	19	22
Contacts received postexposure prophylaxis vaccination	76 (100)	61 (95.3)	60 (100)
Contacts received postexposure prophylaxis antibiotics	76 (100)	61 (95.3)	60 (100)
Contacts with symptoms	22 (28.9)	0	0
Diphtheria cases in inmates and staff			
Symptomatic case-patients that did not receive DAT	0	0	1 (6.7)
Symptomatic case-patients that received DAT	1 (11.1)†	1 (7.7)	0
Asymptomatic carriers	8 (88.9)	12 (92.3)	14 (93.3)
Total	9 (100)	13 (100)	15 (100)
Diphtheria vaccinations			
Inmates	481 (79.6)	240 (94.1)	44 (55.0)
DCS staff	123 (20.4)	15 (5.9)	36 (45.0)
Total	604 (100)	255 (100)	80 (100)

*Values are no. (%) except as indicated. DCS, Department of Correctional Services staff; HCW, healthcare workers. †Died.

Of the 14 correctional services staff contacts, 11 reported mild, nonspecific respiratory symptoms without pseudomembrane or bull neck, and all tested negative by culture and PCR for *C. diphtheriae*. Additional contacts exposed during the index patient’s transport between hospitals included 1 carer, 1 DCS warden, 2 patients (not from the correctional facility), 2 EMS staff, and 4 healthcare workers at the hospital. All were swabbed, vaccinated, and given antimicrobial prophylaxis; all remained asymptomatic. EMS and healthcare workers used PPE during exposure. In accordance with guidelines, those contacts were not isolated but asked to seek healthcare if symptoms started.

By November 13, 2023, a total of 8 (11%) of the contacts tested positive for toxigenic *C. diphtheriae*; they were all inmates and were isolated together. Two of those were experiencing mild nonspecific respiratory symptoms without a pseudomembrane, and the rest were asymptomatic carriers. Sequenced isolates were ST906, a novel lineage not previously described outside of South Africa or the Western Cape.

WCDHW and DCS instituted a vaccination campaign providing Td boosters during November 6–15, 2023. In that week, a total of 604 persons were vaccinated: 123 (51%) of 240 DCS staff (both wardens and healthcare staff at the facility) and 481 (40%) 1,200 inmates (Table). During the vaccination campaign, DCS staff and inmates received health education about diphtheria, its symptoms, risks, and the purpose and possible side effects of vaccination. Some declined the vaccine because of fears of side effects or doubts about its effectiveness. Although transmission risk via fomites is low, DCS staff conducted environmental cleaning, including disinfection of holding cells, laundry, and cutlery affected during the outbreak.

### Outbreak 2: December 2024–January 2025

A second outbreak at the same correctional facility started on December 9, 2024, when the index patient, a young man, experienced symptoms of a sore throat while still at the facility. He was released on December 11 after 9 months of incarceration. On the same day, he sought care at a community-based general practitioner with a sore throat, a typical pseudomembrane, and anterior cervical lymphadenopathy suggestive of the characteristic bull neck. He was admitted to the local tertiary hospital for antimicrobial drugs and timely administration of DAT and made a full recovery. Toxigenic *C. diphtheriae* was subsequently confirmed by culture, Elek testing, and PCR; all sequenced isolates were ST906.

Although the index case was identified on December 11, there was a delay in verifying the correctional facility from which he had recently been released. By December 16, his correctional facility close contacts had been swabbed, started on prophylactic drugs, and isolated. By December 24 their laboratory results were available, identifying 12 additional asymptomatic carriers among the inmates (Table). We identified a total of 64 close contacts from the index patient’s DCS inmates and household contacts; all were asymptomatic, received drugs and were swabbed and vaccinated (Table). WCDHW and DCS strengthened communication through regular situational reports and meetings, and media queries were directed to the relevant departmental spokesperson for a coordinated response. A broader health education and vaccination campaign was conducted. 

### Outbreak 3: February 2025

The third diphtheria outbreak at the facility occurred on February 10, 2025, six weeks after the second outbreak, and was classified as new because of the 1.4-day median incubation of *C. diphtheriae* (range 1–10 days) (*5*). The index patient was a detainee awaiting trial, a young man who had been a close contact of the index patient in outbreak 2 and had declined vaccination at that time. He experienced a sore throat and pseudomembrane on February 10, received DAT, and was confirmed culture positive for toxigenic *C. diphtheriae* on February 16. He recovered fully and returned to the awaiting-trials section.

Prompt contact tracing efforts at the correctional facility during February 13–15 identified 60 asymptomatic detainee close contacts who were immediately isolated together pending results. No household or DCS staff contacts were identified. Contacts were swabbed, provided with antimicrobial drugs, and vaccinated (Table). Initially, 7 (12%) contacts were culture positive; an additional 6 (10%) cases were positive by PCR only. Those 13 cases constituted 22% of contacts; they were isolated together while completing the course of prophylactic treatment and remained asymptomatic throughout the period. Sequenced isolates were all of ST906.

High-resolution core genome phylogeny of ST906 genomes from all 3 outbreaks revealed 2 distinct clusters within the facility (data not shown). The first cluster included genomes from the first outbreak in November–December 2023, whereas the second cluster included genomes from the December 2024–January 2025 and February 2025 outbreaks. The second cluster also included ST906 genomes from sporadic cases and community clusters reported during the same period.

A 2-day campaign was conducted among correctional services staff, providing health education on diphtheria infection prevention and control practices. Among the 70 on-duty DCS staff, 37 (53%) were tested and vaccinated and 20 (29%) accepted antimicrobial prophylaxis only. In total 57/70 (81%) of on-duty staff received prophylaxis, vaccination, or a combination. That wider screening approach identified an additional 2 positive asymptomatic carriers, 2/37 (5%) of DCS staff tested, who were advised to self-isolate while completing treatment. Although facemasks, hand sanitizer, and latex gloves are normally available to DCS staff, the provision of those to visitors to the facility was instituted during outbreak 3.

## Discussion

We report recurrent outbreaks of toxigenic diphtheria that occurred in a correctional facility, requiring intersectoral public health responses that address asymptomatic carriage and waning childhood vaccine–derived immunity. Our analysis describes the public health response to 3 outbreaks of toxigenic *C. diphtheriae* that resulted in a total of 37 cases (3 symptomatic cases and 34 asymptomatic carriers) across both detainee and DCS staff populations and 1 death of an inmate from the awaiting-trials section of a correctional facility. Despite prompt outbreak response, the overcrowding and closed setting led to a substantial number of close contacts in each instance.

Each outbreak was caused by the same sequence type, which was first detected in the Western Cape Province in 2023 both at this facility and among sporadic cases in the community. ST906 appears to be localized in the province as of February 2025 and has not been reported from any other geographic region in South Africa. Both the first and second outbreaks at the facility were caused by new transmission events from the community, whereas the third reported outbreak appears to have been caused by ongoing circulation of the strain within the facility possibly introduced during the second outbreak. We will clarify the sources more precisely in a separate report.

Multiple infectious diseases spread rapidly in prison settings; a systematic review of infectious disease outbreaks across 7 countries found outbreaks of tuberculosis, influenza, measles, mumps, varicella, adenovirus, and SARS-CoV-2 (*1*). Containment of outbreaks is limited by the large number of close contacts and high degree of movement of the awaiting-trial detainees and staff (*1*). Traditional outbreak response tools, isolation and quarantine, are challenging to implement and contribute to delays in court processes (*1*). Approaches relevant to the carceral setting include opt-out testing, empirical vaccination, and requiring full immunization of staff as keys to preventing outbreaks (*13*).

During the second outbreak, infectious disease specialists shortened the deisolation protocol for laboratory-confirmed cases from 14 days to 7 days, if clinical response was good and repeat swabs on days 3–6 were culture negative. That change was made on the basis of evidence of faster time-to-clearance by antimicrobial therapy (4–6 days) (*5*).

Vaccine coverage among 6-year-olds and 12-year-olds in Western Cape is <50%, contributing to the outbreaks in the community and closed settings such as prisons (*14*,*15*). Optimizing coverage in those age groups could reduce risk for diphtheria among young adults.

Outbreak challenges included coordination, contact tracing, and vaccine hesitancy. Security concerns required close collaboration between WCDHW district officials and DCS to protect detainees and staff. A key lesson was the importance of leveraging COVID-19 outbreak response institutional knowledge and relationships, alongside intersectoral collaboration, to enable timely contact tracing, symptom screening, testing, prophylaxis, and vaccination. Ongoing engagement was key in clarifying overlapping responsibilities between stakeholders.

Tracing released detainees, off-duty staff, and unidentifiable court contacts was difficult. We identified no DCS staff as contacts during outbreak 2, possibly because of vaccination in outbreak 1, consistent use of PPE, and a desire to maintain minimum staffing levels during the festive season for continuity of essential services. A limitation of our study was incomplete information on the number of DCS staff and court contacts exposed in each outbreak, which could have affected the estimates of vaccination coverage among staff at the facility.

The presence of asymptomatic carriers makes declaration of the end of a diphtheria outbreak challenging; some asymptomatic carriers can be colonized for up to 18.5 days, which could result in ongoing transmission (*5*). Epidemiologic surveillance can be conducted in prisons by screening inmates and staff for infectious disease as a way to proactively identify asymptomatic cases, although the cost of setting up such programs and providing vaccination needs to be weighed against possible low uptake (*1*). More local research is needed to understand the drivers of low vaccine uptake specifically in correctional facility detainees and staff in a bid to improve vaccine acceptance in future vaccination campaigns.

Globally, diphtheria outbreaks have been documented in community settings as well as refugee centers; mass vaccination efforts have been required to contain such outbreaks (*16*). A suggested alternative approach is mass antimicrobial prophylaxis; modeling studies estimate this approach to be sufficient to contain outbreaks (*5*). Although it is a relatively simple intervention and may be considered if a future outbreak occurs, the cost, feasibility, and benefit of that approach in the correctional facility with a highly mobile awaiting-trial population would need to be considered, along with the risk of antimicrobial resistance. We did not consider that approach appropriate for the subset of prisoners awaiting trial.

By formulating locally relevant clinical guidelines for healthcare workers on antitoxin eligibility, referral pathways, and isolation and deisolation protocols, infectious disease specialists supported the broader outbreak response. Kendig et al. emphasized the vital role of strengthening infection prevention and control practice in prison settings by training staff and inmates on handwashing, ensuring adequate sanitation facilities, and having appropriate outbreak response policies (*17*). The global supply shortage of DAT needed to manage diphtheria cases has also been highlighted during outbreaks, and international health bodies are encouraged to advocate for manufacturers to increase timely access to those medications.

As South Africa rolls out Tdap vaccines, which are more expensive than Td vaccines, the cost of mass vaccination efforts increases (*18*). An estimated 27% of diphtheria outbreaks could be contained by achieving full vaccination coverage alone, whereas simultaneously treating symptomatic cases promptly with antimicrobial drugs would contain 70% of outbreaks (*5*).

Immunization against diphtheria is 97% effective, but immunity wanes after ≈10 years; 30%–60% of adults become susceptible to infection again because the toxoid nature of the vaccine enables bacteria to colonize vaccinated persons without symptoms (*5*). Diphtheria-containing booster vaccination for adults is available in many countries at 10-year intervals, although there is no policy guide for that in South Africa (*19*). Although health education talks supported improved vaccine uptake in the diphtheria outbreaks, we note an increase since the COVID-19 pandemic in literature that documents the challenges of vaccination access and a rise in vaccine hesitancy among the public (*20*). That trend has specific implications for those who live and work in prison settings, where rebuilding trust and credibility in vaccines is necessary to address rising vaccine hesitancy (*21**,**22*).

Outbreaks could be caused by a combination of factors, including asymptomatic carriage, waning childhood-vaccine–derived immunity and insufficient herd immunity within communities. We have described the challenges to effective outbreak response occurring in correctional facilities. We recommend several actions for tackling diphtheria and other infectious diseases: strengthen the implementation of current childhood vaccination programs to improve immunity to vaccine-preventable diseases, adopt communication strategies to combat vaccine misinformation and create awareness about diphtheria and other vaccine-preventable conditions, and develop adult vaccination guidelines that address high-risk congregate settings. Such public health interventions are required to mitigate the risk for future vaccine-preventable disease outbreaks, particularly in closed environments like correctional facilities. 
